# Microcephaly epidemic related to the Zika virus and living conditions in Recife, Northeast Brazil

**DOI:** 10.1186/s12889-018-5039-z

**Published:** 2018-01-12

**Authors:** Wayner Vieira de Souza, Maria de Fátima Pessoa Militão de Albuquerque, Enrique Vazquez, Luciana Caroline Albuquerque Bezerra, Antonio da Cruz Gouveia Mendes, Tereza Maciel Lyra, Thalia Velho Barreto de Araujo, André Luiz Sá de Oliveira, Maria Cynthia Braga, Ricardo Arraes de Alencar Ximenes, Demócrito de Barros Miranda-Filho, Amanda Priscila de Santana Cabral Silva, Laura Rodrigues, Celina Maria Turchi Martelli

**Affiliations:** 1 The Aggeu Magalhães Research Center -FIOCRUZ/PE, Av. Professor Moraes Rego, s/n Cidade Universitária, Recife, Pernambuco CEP 50.740-465 Brazil; 2 Pan American Health Organization, Setor de Embaixadas Norte, Lote 19, Brasília, CEP 70800-400 Brazil; 3 Pernambuco State Health Department, Rua Dona Maria Augusta Nogueira, 519, Bongi, Recife, Pernambuco CEP 50751-530 Brazil; 4 Universidade Federal de Pernambuco, Av. Prof. Moares Rego, 1235, Cidade Universitáia, Recife, Pernambuco CEP 50670-901 Brazil; 5 Universidade de Pernambuco, Avenida Agamenon Magalhães, S/N, Santo Amaro, Recife, Pernambuco CEP 501100-010 Brazil; 6 London School of Hygiene & Tropical Medicine, Keppel Street, London, UK; 7 Universidade Federal de Goiás, Avenida Esperança, S/N, Campus Samambaia, Goiânia, Goiás CEP 74690-900 Brazil

**Keywords:** Zika, Ecological study, Socio-economic, Brazil

## Abstract

**Background:**

Starting in August 2015, there was an increase in the number of cases of neonatal microcephaly in Northeast Brazil. These findings were identified as being an epidemic of microcephaly related to Zika virus (ZIKV) infection. The present study aims to analyse the spatial distribution of microcephaly cases in Recife (2015–2016), which is in Northeast Brazil, and its association with the living conditions in this city.

**Methods:**

This was an ecological study that used data from reported cases of microcephaly from the State Health Department of Pernambuco (August 2015 to July 2016). The basic spatial unit of analysis was the 94 districts of Recife. The case definition of microcephaly was: neonates with a head circumference of less than the cut-off point of −2 standard deviations below the mean value from the established Fenton growth curve. As an indicator of the living conditions of the 94 districts, the percentage of heads of households with an income of less than twice the minimum wage was calculated. The districts were classified into four homogeneous strata using the K-means clustering algorithm. We plotted the locations of each microcephaly case over a layer of living conditions.

**Results:**

During the study period, 347 microcephaly cases were reported, of which 142 (40.9%) fulfilled the definition of a microcephaly case. Stratification of the 94 districts resulted in the identification of four strata. The highest stratum in relation to the living conditions presented the lowest prevalence rate of microcephaly, and the overall difference between this rate and the rates of the other strata was statistically significant. The results of the Kruskal-Wallis test demonstrated that there was a strong association between a higher prevalence of microcephaly and poor living conditions. After the first 6 months of the study period, there were no microcephaly cases recorded within the population living in the richest socio-economic strata.

**Conclusion:**

This study showed that those residing in areas with precarious living conditions had a higher prevalence of microcephaly compared with populations with better living conditions.

## Background

Zika virus (ZIKV) is a *Flaviviridae* that can currently be characterized as one of the most significant emerging arboviruses in the Americas considering how widespread its infection has become. The first reports of the circulation of this virus go back to 1947 in Uganda among non-human primates. The first reported human case dates back to 1954 in Nigeria [1, 2].

The first recorded outbreak of the virus outside of the continent of Africa occurred on the Yap Islands in Micronesia in 2007, which was followed by outbreaks in French Polynesia in 2013 and 2014 [3, 4]. During these outbreaks, the disease was identified as being characterized mainly by a rash and arthralgia. The Zika infection was associated with Guillain-Barré syndrome in the adult population of French Polynesia [5].

The circulation of ZIKV was first reported in South America in 2014 [6]. Towards the end of 2014 and beginning of 2015, several cities in Northeast Brazil reported an outbreak of an exanthaematous disease that was clinically different from dengue. The first confirmed case of ZIKV infection in the country occurred in March 2015 [7].

Starting in August 2015, there was an increase in the number of cases of neonatal microcephaly in Pernambuco and throughout other states in Northeast Brazil compared to previous patterns [8]. This region aggregated approximately 90% of the 1501 livebirths that were registered as having microcephaly, which was investigated from November 2015 to February 2016 [9]. This epidemic of microcephaly of an unknown aetiology was promptly declared as being a national public health emergency by the Brazilian Ministry of Health in November 2015 [10]. These space and time clusters of microcephaly were described in series of cases studies reporting on neonates with a foetal brain disruptive sequence, a previously rare phenotype involving microcephaly and cerebral abnormalities, who also had presumptive or confirmed in utero ZIKV infection [11, 12]. Subsequently, a causal relationship was established between ZIKV infection and congenital ZIKA syndrome, which includes microcephaly and other abnormalities [13–15].

The transmission of vector-borne diseases is currently a public health challenge in urban areas [16]. Previous studies on dengue conducted in the city of Recife in Northeast Brazil reported a variable disease prevalence that was based on the place of residence. This is a reflection of the poor living conditions: overcrowding, high mobility and high exposure to the infected vectors [17]. This same socio-economic pattern has been seen in Bancroftian filariasis, which was a major endemic public health problem for several decades [18–20]. There are important social inequalities that exist in the inner city, but universal access to healthcare is warranted by a public health system [21, 22].

Within the context of the Zika epidemic, there is a lack of information regarding the relationship between the occurrence of microcephaly and the socio-economic conditions in the urban environment. The present study analyses the spatial distribution of the microcephaly cases in the epidemic in Recife (2015–2016) and its association with living conditions.

## Methods

**Fig. 1:**
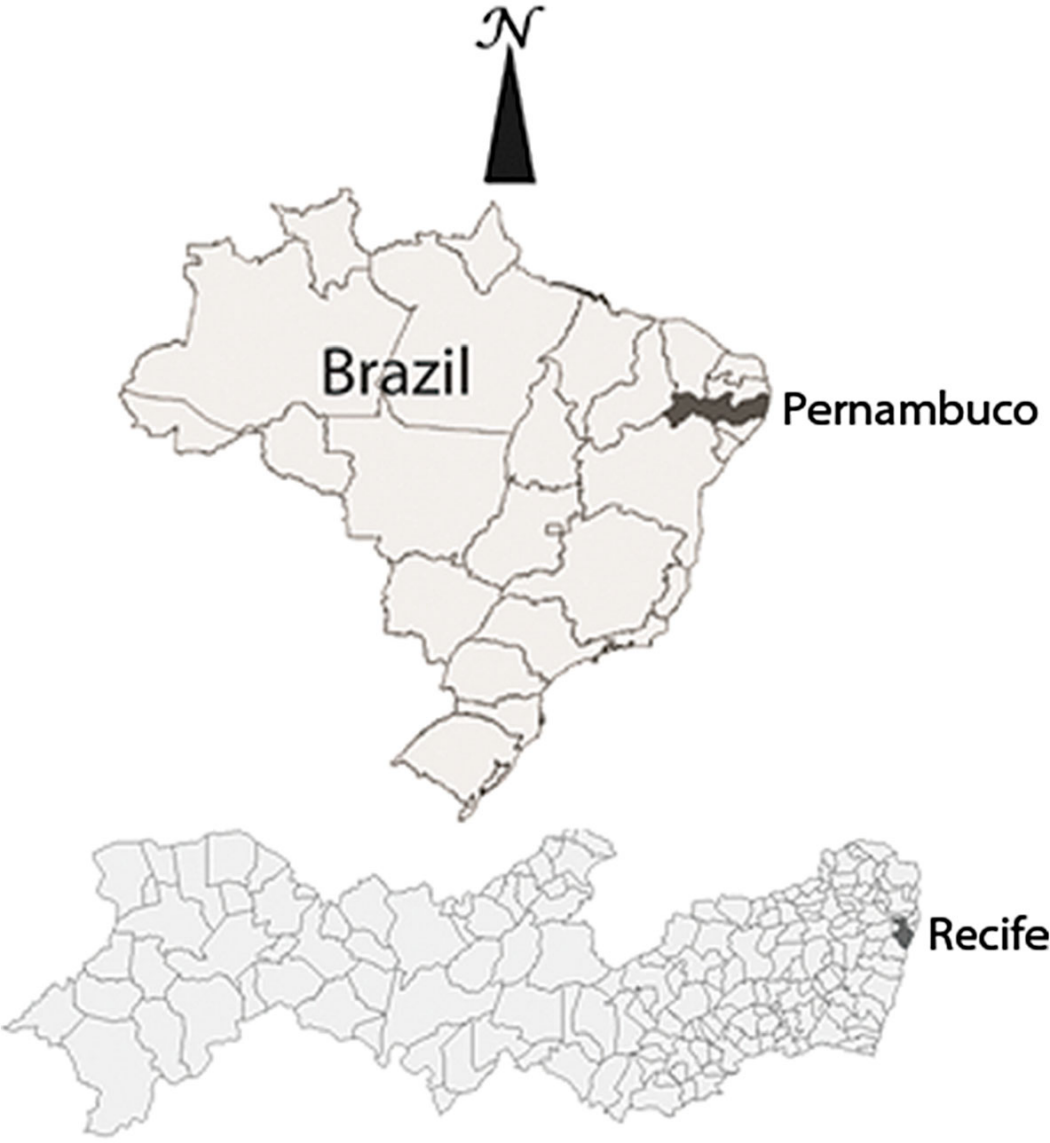
Location of the capital city of Recife, Pernambuco State, Brazil

This study was conducted in the city of Recife in Northeast Brazil. The city is located on the Atlantic coastal plain (8^°^03’S 34^°^52’W), has an area of 218 km^2^ and is divided into 94 districts. It has a population of 1.5 million (Fig. 1). This implies that it has a population density of approximately 7000 inhabitants per square kilometre, which is one of the highest in Brazil. The human development index for the city was 0.772, and only 69% percent of the population was connected to the sewage system in 2010 [21].

This is an ecological study that uses data from reported cases of microcephaly during a one-year period from 02/08/2015 to 31/07/2016. These data were provided by an electronic surveillance system that was setup by the Ministry of Health and the State Health Department of Pernambuco. The cases are reported by the health services professionals in this real time system, generating a database managed by this State Department. The district was the basic spatial unit of analysis in this study.

For the present study, a case definition of microcephaly refers to those neonates with a head circumference of less than the cut-off point of −2 standard deviations (SDs) below the mean value from the established Fenton growth curves according to sex and gestational age. The choice of the −2 SD cut-off was due to its higher sensitivity for screening suspected microcephaly cases compared with the −3 SD cut-off, which is a more specific choice.

The Brazilian minimum wage in December 2016 was 880.00 BRL, which is equivalent to approximately 260.00 USD [23]. As an indicator for the living conditions, the percentage of heads of households with an income of less than twice the minimum wage (1760 BRL/month), including head of households with no income, was calculated for each district using data from the 2010 census. The city of Recife has a diverse ethnic population mainly composed by European, Black Africans and Indigenous descendents. The majority of the residents are non-white/black that has lower income compared to the white population, according to demographic census [24]. Subsequently, the 94 districts of the city of Recife were classified into four homogeneous strata in terms of living conditions using the K-means clustering algorithm [25]. This technique allows for the identification of homogeneous groups of districts according to the indicator used. It establishes intra-strata homogeneity and between-strata heterogeneity. Statistically, this aims to maximize the variance between the groups and minimize the variance within each group.

The microcephaly cases were georeferenced with QGIS software (QGIS Development Team, 2015. QGIS Geographic Information System. Open Source Geospatial Foundation.

https://qgis.org/en/site/) through the geocoding tool that transforms registered addresses into a tabular database for the database of sites stored in QGIS and returns the result in the form of geographical coordinates (latitude and longitude) for all of the addresses. The cartographic basis for the city of Recife was provided by the Brazilian Institute of Geography and Statistics (IBGE) website in the shapefile format in the “geographic” projection system (latitude and longitude) and SIRGAS 2000 [26], which was updated in 2010.

First, we produced a thematic map of the living conditions. Second, we plotted the residential location of each of the microcephaly cases over a layer of living conditions.

It should be noted that this map was made at a scale of 1: 100,000, which produces an error of approximately 20 m on the real scale (0.2 mm on the map). Therefore, the residence of each case was located in a broad circle of approximately 1250 m^2^ in a highly urbanized city. Consequently, the precise location of each residence was not possible in this mapping procedure and ethical concerns are not applicable.

To analyse the prevalence of microcephaly among the districts according to the different strata of living conditions, we used the non-parametric Kruskal-Wallis test for independent samples.

## Results

The stratification of the 94 districts according to the percentage of heads of households with a monthly income of less than twice the minimum wage, from which four strata were identified (Table 1).

**Table 1:** Districts and populations of Recife, Brazil in 2010 stratified according to the living conditions

Strata	Districts (n)	Population	%	Strata Centre^a^
1	14	205,926	13.5	22.2
2	10	100,351	6.6	40.0
3	30	533,540	35.1	68.1
4	40	680,762	44.8	88.0
Total	94	1,520,579	100	–

^a^Final strata Centres (% of heads of households with an income below twice the minimum wage) in the K-means analysis; df = 3; *F* = 570.6; *P* < 0.0001

We performed the K-means procedure with the ANOVA test, and the F value was higher for 4 strata (*F* = 570.6; df = 89) compared to 3 strata (*F* = 523.4; df = 90) and 5 strata (*F* = 527.3; df = 88).

The mean per capita monthly incomes of the populations observed for the strata were 2868.88 BRL (1), 1616.73 BRL (2), 674.76 BRL (3) and 340.70 BRL (4).

**Fig. 2:**
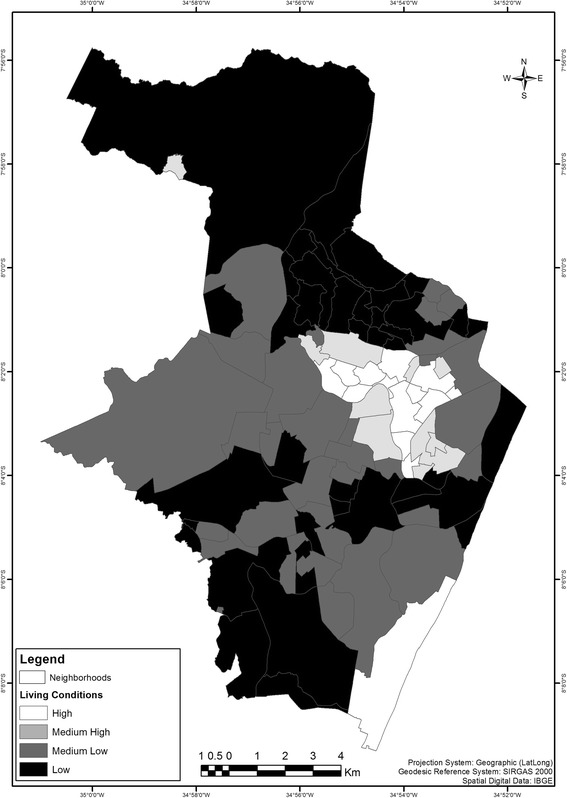
Neighbourhoods and living conditions of Recife, Northeast Brazil, according to the 2010 census data

Figure 2 presents the Recife neighbourhoods aggregated according to the strata of living conditions.

During the study period, 347 suspected cases of microcephaly were reported, of which 142 (40.9%) fulfilled the study definition of a microcephaly case.

Table 2 presents the number of microcephaly cases, the prevalence rates calculated for each stratum of living conditions and the results for the Kruskal-Wallis test for association.

**Table 2:** Prevalence rates of microcephaly stratified by the living conditions in Recife, Brazil

Strata	Microcephaly Notified	Microcephaly Cases	Newborns	Prevalence per 10,000 newborns	95% CI
1	10	3	2487	12.1	2.5–35.2
2	21	8	1290	62.0	26.8–121.8
3	116	55	7984	68.9	51.9–89.6
4	200	76	11,417	66.6	52.5–83.3
TOTAL	347	142	23,178	61.3	51.6–72.2

Kruskal-Wallis test: χ^2^ = 10.94; *p* = 0.012

The highest socio-economic stratum presented the lowest microcephaly prevalence rate, and the overall difference between this rate and the rates of the other strata was statistically significant. The stratum of the higher living conditions had 3 microcephaly cases in 2487 live births, which is compared to 139 cases in 20,691 live births for the other strata. This implies that there was a prevalence ratio (PR) of 5.6 (95% CI: 1.8 to 17.5).

**Fig. 3:**
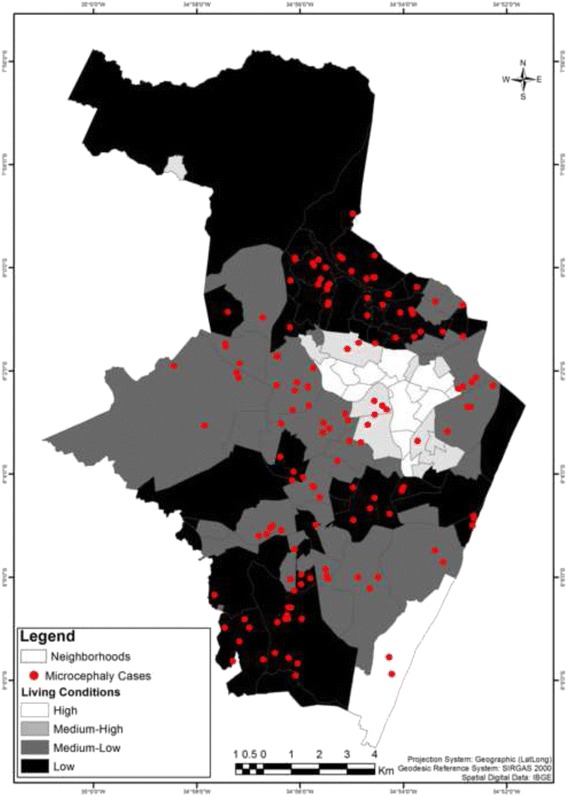
Microcephaly cases and living conditions of the residents of Recife, Brazil

Figure 3 illustrates the results above, presenting the low occurrence of microcephaly cases in the more-privileged districts of the city.

These results indicate that there is a strong association between a higher prevalence of microcephaly and poor living conditions.

Moreover, it was observed that microcephaly cases occurred in only 2 of the 14 districts of stratum 1 and in 4 of the 10 districts of stratum 2. In addition, all of the cases that occurred in strata 1 and 2 did so during the first 6 months of the epidemic (August 2015 to January 2016), while in strata 3 and 4, 27% of the cases (35 out of 131) occurred between February 2016 and July 2016.

## Discussion

In the present study, we showed that residing in areas with precarious living conditions was associated with a higher prevalence of microcephaly compared to residing in areas with better living conditions.

The causal relationship between congenital ZIKV infection and microcephaly has become evident [13–15], but the association between microcephaly and living conditions has not yet been explored. We used a framework of socio-economic conditions to depict the distribution of microcephaly cases in the city of Recife. The strata of socio-economic conditions showed that approximately 40% of the urban population of the city of Recife lives in districts where the heads of households earn a low monthly income. The evaluation of strata by living conditions revealed small wealthy inner-city areas surrounded by large, populated deprived areas, except for the more recently urbanized southern area located along the coastline. The city of Recife has a high population density and is crossed by two rivers. The year-round temperature and humidity levels combined with clusters of inadequate houses built in overcrowded areas without basic sanitation provide an ideal environment for the transmission of vector-borne diseases such as filariasis and dengue and now Zika and Chikungunya [18, 20, 27]. Within the global scenario, increased amounts of unplanned urbanization has transformed the urban areas of poor and middle-income countries into breeding grounds for vector-borne diseases [16]. The discussion of social and living condition inequalities and the consequent social determinants of health highlights important issues related to infectious and non-infectious diseases [22, 28].

It should be mentioned that the analysis of the spatial distribution of microcephaly cases was performed shortly after the first wave of the ZIKV infection in Recife, a densely populated city in Northeast Brazil. The point pattern map of microcephaly cases shows that the bulk of microcephaly cases related to the epidemic were in deprived areas. Only three out of the 142 cases were located in the stratum of the wealthiest district and were identified in the first months of the epidemic. The interpretation of this finding is complex. In Brazil, abortion is legal only in cases for which there is risk to the mother’s life and in cases of rape. Our study does not allow for the evaluation of whether women with better socio-economic conditions have easier access to abortion and other ways for preventing Zika virus exposures.

In the study area, after almost 30 years of dengue virus circulation, there is still a significant association between dengue seroprevalence and the living conditions in the city of Recife [17]. The population living in low-income areas was more prone to previous dengue infection (91.1%) than that in areas with better living conditions (74.3%) according to a large survey conducted in 2010 [17]. Nevertheless, according some authors [29], dengue transmitted by *Aedes aegypti* is not strictly considered a disease of poverty, which is based on outbreaks in cities that could be described as wealthy.

One limitation of our study is that the case definition of microcephaly refers only to the head circumference measurement of neonates and does not take into account other clinical and/or laboratory assessments. However, this was the notification criteria used during the study period. Furthermore, it should be noted that the adopted criterion (2 SDs) differs slightly from that previously used (below the third percentile of the Fenton Curve). Thus, it approaches the conditions established by the Intergrowth method, which is currently recommended. Our findings are also prone to the limitation that is inherit in ecological studies, and the spatial unit of analysis used may not be homogenous in terms of the socio-economic conditions. In addition, the number of cases was insufficient for more stratified analyses.

Our findings raise an interesting hypothesis about the spatial heterogeneity of microcephaly occurrence related to living conditions. We are aware that our findings are the first that resulted from using an approach with an ecological design. Exploring the impact of living conditions and other factors should be taken into account to explain the risk differences of microcephaly events at the individual level in epidemic and post-epidemic contexts.

## Conclusion

Our research highlights that residing in urban areas with precarious living conditions is associated with a higher prevalence of microcephaly compared with residing in areas with better living conditions.

Monitoring the spatial distribution of microcephaly cases and its correlation with living conditions, in addition to seroprevalence surveys for circulating arbovirus, could be a valuable tool for the assessment of the dynamics of intra-urban disease transmission. The overlap of the layers of disease occurrence and deprived areas is useful for public health interventions such as vector control.
